# Fermented black soldier fly larvae as a sustainable replacement for marine fish in Asian swamp eel diets

**DOI:** 10.14202/vetworld.2025.1002-1013

**Published:** 2025-04-25

**Authors:** Yifan Xiang, Shaoqi Gao, Yanhui Luo, Gaojian Tang, Xiuwei Zou, Kai Xie, Wenjie Niu, Xinyi Li, Junan Xiang, Ling Zhang, Zhu Tan, Xiaoyu Zeng, Bo Wang

**Affiliations:** 1 State Key Laboratory of Animal Nutrition and Feeding, Department of Animal Nutrition and Feed Science, College of Animal Science and Technology, China Agricultural University, Beijing, China; 2 Hunan Airbluer Environmental Protection Technology Co., Ltd., Hunan Changsha, China; 3 Laboratory of Aquatic Animal Nutrition and Feeding, Department of Animal Nutrition and Feed Science, Hunan Agricultural University, Hunan, China

**Keywords:** Asian swamp eel, black soldier fly larvae, fermented insect protein, hepatic lipid accumulation, intestinal immunity, sustainable aquafeed

## Abstract

**Background and Aim:**

Fermented black soldier fly larvae (BSFL) have emerged as a sustainable and economically viable protein source in aquaculture. However, their potential as a replacement for marine fish in the diets of Asian swamp eels (*Monopterus albus*, ASEs) remains underexplored. This study assessed the effects of partially substituting marine fish with fermented BSFL on ASE growth performance, intestinal development, and hepatic health.

**Materials and Methods:**

A total of 480 ASEs were randomly assigned to four dietary groups: control (40% marine fish), BSFL34 (13.4% BSFL), BSFL61 (24.1% BSFL), and BSFL82 (32.8% BSFL), replacing marine fish on a dry matter basis. All diets were isonitrogenous and isoenergetic. Fish were reared in net cages for over 90 days, and parameters including survival rate, growth metrics, muscle and liver histology, intestinal morphology, gene expression (quantitative real-time polymerase chain reaction), and inflammatory protein levels (Western blotting) were assessed.

**Results:**

Survival rate was significantly higher in the BSFL61 group (p < 0.05). Growth performance was not impaired across BSFL-fed groups, although BSFL61 showed reduced body weight compared to BSFL82 (p < 0.05). Muscle fiber size, satellite cell number, and muscle triglyceride (TG) content remained unchanged. BSFL82 showed increased hepatic TG accumulation (p < 0.05) and reduced liver fibrosis, while BSFL61 exhibited a significantly lower hepatosomatic index and increased fibrosis. Intestinal villus height was reduced in BSFL34 and BSFL61, while goblet cell density increased in all BSFL groups. Notch1 expression was upregulated in BSFL61 and BSFL82, whereas ctnnb1 and wnt5a were downregulated. Inflammatory markers nuclear factor-kappa B and interleukin-1 beta were elevated in BSFL-fed groups, indicating an activated mucosal immune response.

**Conclusion:**

Partial replacement of marine fish with fermented BSFL enhanced ASE survival, modulated intestinal immunity, and improved mucosal barrier function, without compromising overall growth performance. However, excessive inclusion may induce hepatic lipid accumulation and affect intestinal morphology. These findings support the use of fermented BSFL as a sustainable aquafeed ingredient, though inclusion levels should be carefully optimized to balance health benefits and growth efficiency.

## INTRODUCTION

The Black Soldier Fly Larvae (*Hermetia illucens*, BSFL) are recognized as efficient bioconverters capable of transforming organic waste into valuable resources for animal feed. They exhibit rapid and effective decomposition of various organic substrates, including kitchen waste [1], human and animal feces [2–5], and plant residues [6]. The nutritional composition of BSFL is largely influenced by the type of substrate and developmental stage [7, 8]. On average, BSFL contain approximately 42% crude protein (CP) and 29% fat, depending on rearing conditions [9]. These characteristics make BSFL a suitable feed component for a wide variety of livestock, including poultry [10, 11], swine [12], and fish species [13, 14].

The Asian swamp eel (*Monopterus albus*, ASE) is a commonly consumed freshwater species in China [15] and Southeast Asia [16–18], valued for its nutritional profile and palatability [19]. As a carnivorous species, ASE feeds on small fish [20], insects [21], earthworms [22], snails [21], and other live prey. In China, it is common practice for farmers to provide ASE with pelleted compound feed supplemented with minced fish. However, the escalating cost of fish [23, 24] has prompted a search for more affordable alternatives [25–27]. BSFL has emerged as a promising aquaculture feed ingredient due to its high protein content, cost-effectiveness, reproductive efficiency, and ease of production management [8, 28]. Studies have shown that BSFL-based diets support good digestibility in Atlantic salmon [29] and promote satisfactory growth in ASE and Siberian sturgeon [30,31]. Moreover, replacing fish oil with BSFL oil in rainbow trout diets has been shown to improve growth performance and meat quality [32]. In addition, BSFL contains bioactive compounds that enhance antioxidant activity and immune responses in European seabass [28].

Our field observations in Hunan Province, China, revealed that marine fish are frequently incorporated into ASE diets. However, issues such as variable species composition, inconsistent quality, logistical difficulties related to cold-chain storage, and marine pollution necessitate alternative feed solutions with more consistent nutritional profiles. Accordingly, BSFL may serve as a viable replacement for marine fish in ASE diets. Traditional drying methods for BSFL involve high temperatures, which can degrade nutrients [33, 34] and bioactive properties [35]. Although fresh or frozen BSFL retain these qualities more effectively, they require cold-chain logistics, raising costs significantly. Fermentation offers a more economical method for preserving BSFL [36, 37], enabling storage and transportation at ambient temperatures.

In addition to supplying protein and fat, BSFL contains antimicrobial agents such as medium-chain fatty acids (MCFAs) [38, 39] and antimicrobial peptides (AMPs) [40–42]. Our recent findings demonstrated that methanol extracts from BSFL possess potent anti-Gram-positive bacterial activity [43]. Fermentation protects the nutritional and functional compounds in BSFL from heat degradation [44], while also generating novel bioactives such as lactic acid [45]. This process enhances the content of unsaturated fatty acids and essential amino acids [46], improves antimicrobial activity, and extends shelf life [47].

While BSFL have been widely recognized as a sustainable alternative protein source in aquafeeds, existing research has primarily focused on their application in conventional species such as tilapia, salmon, and seabass. Although some studies have explored the inclusion of BSFL in the diets of ASE and other carnivorous fish, these investigations often rely on dried or unfermented BSFL and fail to address the challenges associated with nutrient preservation and long-term storage. Furthermore, limited attention has been given to the use of *fermented* BSFL, which may offer additional nutritional and immunomodulatory benefits while mitigating storage and transport issues. Despite the increasing adoption of marine fish in commercial ASE diets in regions such as Hunan Province, China, the implications of substituting marine fish with fermented BSFL on ASE growth, intestinal morphology, and hepatic health remain poorly understood. There is a pressing need for experimental data to inform the formulation of nutritionally balanced and cost-effective diets that align with sustainable aquaculture practices.

This study aims to evaluate the potential of fermented BSFL as a partial replacement for marine fish in the diet of ASE *(M. albus*). Specifically, the research investigates the effects of different substitution levels of fermented BSFL on growth performance, muscle development, intestinal structure and immune response, and hepatic lipid metabolism. By examining both physiological and molecular responses, the study seeks to determine optimal inclusion levels that promote health and survival without compromising growth efficiency. The findings are intended to inform the development of sustainable, economical, and nutritionally viable feed formulations for ASE and potentially other aquaculture species.

## MATERIALS AND METHODS

### Ethical approval

This study has received research ethics approval from the Institutional Animal Care and Use Committee at China Agricultural University, with approval number AW92704202-1-1.

### Study period and location

This study was conducted from August 1 to October 1, 2022, in Yuanjiang Cao Wei Agricultural Machinery Professional Co-operative.

### Fermentable bacteria

The lactic acid bacterium *Lactobacillus agilis* used in this study was obtained from ATCC (JCM 1050, ATCC 43616). Several experimental procedures, including BSFL fermentation, diet preparation, animal feeding, and sample collection, have been previously described in detail in our earlier publication [48].

### BSFL fermentation and experimental diet preparation

BSFL were reared on a substrate composed of deodorized kitchen waste and wheat bran and harvested at the fifth instar stage. The larvae were then minced using a Feed Grinding Machine (HF-360, Haichuan Machinery Factory, Wenzhou, China). Per kilogram of BSFL, the minced material was mixed with 80 g of glucose, 80 mL of L. agilis (5 × 10^5^ colony forming units/mL), and 100 g of rice bran. The final moisture content (MC) was adjusted to 72%. This mixture, referred to as BSFL fermented homogenate (BSFFH), was sealed in fermentation bags and incubated at room temperature (approximately 25°C) for 72 h. The fermented product developed a yogurt-like aroma and reached a pH below 4.2, after which it was refrigerated at 4°C until further use.

Feed formulations, including compound feed, marine fish, and BSFFH, were prepared according to the Association of Official Analytical Chemists (AOAC) International standards [49]. CP was analyzed using a Kjeldahl Nitrogen Protein Analyzer (RapidNIII, Elementar, Frankfurt, Germany). Crude fat (ether extract, EE) was assessed using an Automatic Fat Analyzer (XT10i, ANKOM, Wayne, USA). Crude ash (CA) was determined by combusting samples in a muffle furnace (STM-8-12, SAFTherm, Luoyang, China) at 550°C. Gross energy (GE) was measured using an Oxygen Bomb Calorimeter (C 6000 global, IKA, Cologne, Germany). MC was determined by oven drying at 105°C using an Electrothermal Thermostatic Drying Oven (DHG-9037A, Jinghong, Shanghai, China).

In the dietary treatments, chilled marine fish (mainly *Trichiurus lepturus* and *Larimichthys polyactis*) were replaced with BSFFH on a dry matter basis, while maintaining similar CP and GE levels across groups. The control diet consisted of 60% compound feed and 40% marine fish. In the BSFL34 group, 13.4% BSFFH replaced part of the fish component, resulting in 60% compound feed, 26.6% marine fish, and 13.4% BSFFH. The BSFL61 group included 60% compound feed, 15.9% marine fish, and 24.1% BSFFH, while the BSFL82 group contained 60% compound feed, 7.2% marine fish, and 32.8% BSFFH. The compound feed (Tech-Bank Feed Industry Co., Ltd., Ningbo, China) comprised 60% fish meal, 22% starch, 4% brewer’s yeast meal, 4% soybean meal, and 10% additional ingredients such as vital wheat gluten, multivitamins, multiminerals, and food additives.

Marine fish were homogenized before mixing with BSFFH and compound feed. All diets were weighed to ensure consistent feeding quantities. BSFFH was stored at 4°C, marine fish at −20°C, and compound feed at ambient temperature. Diets were formulated and administered after returning all ingredients to room temperature. Experimental conditions were consistent across all groups except for diet composition.

Given the limited research on ASE nutritional requirements, diets were designed based on local aquaculture practices and literature sources [50–52]. The control group received 60% compound feed and 40% marine fish. CP levels were equalized across all treatment groups. Diet compositions were based on ingredient nutrient profiles (Table 1), and the corresponding nutrient values are presented inTable 2.

**Table 1:** Main nutrients of dietary ingredients (Dry matter base).

Ingredients	CP/%	Crude lipid (EE/%)	CA/%	GE/MJ/kg	Calcium (%)	Phosphorus (%)	MC/%
Compound feed	44.53	4.87	10.95	18.54	3.40	1.89	0
Marine fish	68.19	7.77	16.67	19.40	4.83	2.99	82.00
BSFFH	45.00	18.39	10.32	19.61	3.35	0.81	72.00

Compound feed was purchased from the Ningbo Tianbang Feed Technology Co. BSFFH=Black soldier fly larvae fermented homogenate, CP=Crude protein, EE=Ether extract, CA=Crude ash, GE=Gross energy, MC=Moisture content

**Table 2:** Formula for experimental diets of different groups (Wet base).

Ingredients	0%	34%	61%	82%
BSFFH/%	0	13.4	24.1	32.8
Marine fish percentage	40.0	26.6	15.9	7.2
Compound feed^1^/%	60.0	60.0	60.0	60.0
Nutrient content of feed				
CP/%	31.6	31.7	31.7	31.7
EE (Crude lipid)/%	3.48	3.98	4.39	4.71
CA/%	7.77	7.76	7.74	7.73
Calcium/%	2.39	2.40	2.40	2.41
Phosphorus/%	1.40	1.31	1.27	1.25
GE/MJ/kg	12.52	12.79	12.95	13.18

CP=Crude protein, EE=Ether extract, CA=Crude ash, GE=Gross energy, BSFFH=Black soldier fly larvae fermented homogenate.

1 Obtained from Qinfeng Feed Industry Co., Ltd. (Jiangsu, China)

### Experimental animals and design

Before stocking, nets were disinfected using a 500:1 diluted iodine solution. A total of 480 ASEs, each weighing approximately 50 g, were obtained from a local hatchery. The eels were placed in net cages (2 m × 2 m × 2 m) covered with *Alternanthera philoxeroides* to provide a climbing substrate [49]. The nets were installed in a 300 m^2^ pond equipped with running water, a filtration system, and aeration.

ASEs were randomly assigned to four groups: Control, BSFL34, BSFL61, and BSFL82, with six replicates per group and 20 fish per net. Following a two-week acclimatization period on the control diet, feeding was conducted once daily between 17:00 and 18:00 at 3%–4% of body weight. Feed consumption was monitored to ensure that it was completed within 20 min, and feeding amounts were adjusted accordingly.

Fish were fasted for 24 h at the end of the acclimation period. The feeding trial lasted 90 days. BSFL inclusion did not affect pond water quality, as indicated by stable parameters shown in Table 3 (pH 7.6–7.8, ammonia-nitrogen <0.5 mg/L, nitrite <0.05 mg/L, and dissolved oxygen >6.0 mg/L).

**Table 3:** Water quality parameters.

	Beginning	One month after feeding	p-value
pH	7.76 0.18	7.76 0.10	0.832
Ammonia and nitrogen (mg/L)	0.13 0.03	0.16 0.01	0.163
Nitrite (mg/L)	0.016 0.012	0.014 0.004	0.801
Dissolved oxygen (mg/L)	7.83 1.79	8.97 1.79	0.481
Temperature (℃)	31.67 0.65	32.20 0.92	0.457

### Sample collection and tissue processing

At the end of the trial, fish from each net were counted to calculate survival rates. Body weight and length were measured. Six fish per net were randomly selected and euthanized through percussive stunning. Liver, dorsal muscle, and hindgut samples were collected. Each tissue was divided: One part was fixed in 4% paraformaldehyde for 48 h, while the other was snap-frozen in liquid nitrogen and stored at −80°C.

Fixed tissues were washed, dehydrated, and embedded in paraffin. Sections were cut at 5 µm thickness. Liver samples were stained with hematoxylin-eosin (H&E) and picrosirius red; muscle samples with H&E and immunofluorescence; and intestinal samples with H&E and periodic acid-Schiff (PAS) stains.

### Immunohistology staining

Paraffin sections were deparaffinized, rehydrated, and subjected to antigen retrieval in sodium citrate buffer (10 mM trisodium citrate, 0.05% Tween-20, pH 6.0) at 95°C–100°C for 20 min. Sections were then blocked with 10% goat serum and 0.5% Triton X-100 in Tris Buffered Saline (TBS) for 1 h, followed by incubation with primary antibody for 12 h and secondary antibody for 1 h. Anti-PAX7 antibody was sourced from Developmental Studies Hybridoma Bank (DSHB) (Iowa City, USA), and fluorescein isothiocyanate (FITC) goat anti-rabbit immunoglobulin G (H+L) from ABclonal (Wuhan, China). Both antibodies were diluted 1:200.

### PAS staining

Sections were deparaffinized, hydrated, and oxidized in 0.5% periodic acid for 10 min. After washing with distilled water, they were stained with Schiff reagent for 1 h, counterstained with hematoxylin, dehydrated, and mounted.

### Picrosirius red staining

Sections were deparaffinized, rehydrated, and stained in Picrosirius red for 1 h, followed by two washes with 0.5% acetic acid. The samples were then dehydrated and mounted in resin.

### Quantitative real-time polymerase chain reaction (qRT-PCR)

Total RNA was extracted from dorsal muscle and posterior intestine using Beyozol reagent (R0011, Beyotime, Shanghai, China). RNA was reverse-transcribed into complementary DNA (cDNA) using the BeyoRT™II First Strand cDNA Synthesis Kit (RNase H minus, D7168S, Beyotime). qRT-PCR was performed using ChamQ SYBR qPCR Master Mix (Without ROX, Q321-02, Vazyme, Nanjing, China) on a CFX RT-PCR system (MyiQ2, Bio-Rad). Expression was normalized to 18S ribosomal RNA. Primer sequences are listed in Supplementary Table 1.

**Supplementary Table 1:** Primers used for qRT-PCR (qPCR) in trial.

Used for	Gene names	Gene bank accession no.	Primer sequence (5′-3′)
qPCR	*pa×7* ^1^	XM_020622681.1	F: CATTGATGGCCTAGCGGTTG
			R: GGGAAAATGTGTGCTGTCGG
	*myf5* ^2^	XM_020613678.1	F: GCAATTCAGAGGCAGCAGTGAG
			R: ACTGGAGGCAATGTCCTGGCT
	*myod* ^3^	XM_020593504.1	F: CTCCGAAACTCCAAACGGTGG
			R: GGTGTGGCAGGATGTTCAGGT
	*myog* ^4^	XM_020592886.1	F: TCGGAGAGCGGCAACATTGAG
			R: TCGCTTGACGACGACACTCTG
	*mrf4* ^5^	XM_020613683.1	F: ACCCAAGGTGGAGATTTTACGCAG
			R: GAGGACTCACTGGTTTCTTCTCTC
	*lgr5* ^6^	XM_020613539.1	F: CTGGTGCTGCGCTCTGTTGAT
			R: TGACTCGGGGCAGGTCTTCTT
	*notch1* ^7^	XM_020621119.1	F: CAGCGTCCTCCACACCAATGT
			R: CCAGGTACACCACAGACCCTT
	*ctnnb1* ^8^	XM_020596887.1	F: GGCTACAGACAGGAAGACCCA
			R: AACCAGGCCAGTTGGTTGGAG
	*ctnnb2* ^9^	NM_001001889.1	F: CCAAGGCAGCAGGAGCACTTC
			R: AAATGGCGGCGGACACATCAC
	*wnt5a* ^10^	XM_020620705.1	F: CTCACACTGGTCACGCTCCTTATG
			R: CAGAGGCTGGGCACCAATGATG

1 Paired box 7,

2 Myogenic factor 5,

3 Myogenic differentiation factor,

4 Myogenin,

5 Myogenic factor,

6 Leucine rich repeat containing G protein coupled receptor 5,

7 Notch receptor 1,

8 Catenin beta 1,

9 Catenin beta 2,

10 Wingless-type MMTV integration site family member 5a. qRT-PCR=Quantitative real-time polymerase chain reaction

Muscle development genes (pax7, myf5, *myod*, myog, and mrf4) and intestinal/goblet cell differentiation markers (lgr5, notch1, notch2, ctnnb1, and wnt5a) were assessed.

### Western blotting

Total proteins were extracted using radioimmunoprecipitation assay buffer (10 mM Tris, 150 mM sodium chloride (NaCl), 10 mM potassium chloride, 1 mM ethylenediaminetetraacetic acid, pH 7.4) containing protease inhibitors (0.5 mM phenylmethylsulfonyl fluoride, 100 mM sodium fluoride, and 1 mM sodium orthovanadate). Proteins (1 µg/µL) were mixed with Sodium Dodecyl Sulfate (SDS) sample buffer, boiled at 95°C for 5 min, and resolved by SDS-polyacrylamide gel electrophoresis (10% or 12%) at 65 V for 40 min and 115 V for 60 min. Proteins were transferred to polyvinylidene fluoride membranes at 100 V for 90 min in transfer buffer (25 mM Tris-HCl, 192 mM glycine, 20% methanol, pH 7.6).

Membranes were blocked in 5% skim milk (Tris-Buffered Saline with Tween-20, TBST) for 1 h, incubated with primary antibody (1:1000) in 5% bovine serum albumin-TBST overnight at 4°C, followed by secondary antibody incubation (1:1000) for 1 h at room temperature. Bands were visualized using Enhanced Chemiluminescence (ECL) (P0018FS, Beyotime), imaged with a Tanon 5200 system (Shanghai, China), and analyzed using ImageJ (NIH, USA). Antibodies against nuclear factor-kappa B (NfκB) (AF5243), phospho-NfκB (AF5875), and interleukin-1 beta (IL-1β) (AF7209) were purchased from Beyotime Biotech (Shanghai, China); β-Actin (bs-0061R) was sourced from Bioss Inc. (Beijing, China).

### Triglyceride (TG) analysis

Approximately 50 mg of liver or muscle was homogenized in a 2:1 chloroform: methanol mixture, centrifuged at 5000× *g* (4°C, 10 min), and washed with 0.9% NaCl. After separation at 400× *g*, the chloroform phase was collected, freeze-dried, resuspended in 2-propanol, and analyzed for TG content using a commercial assay kit (A110-1-1, Nanjing Jiancheng Bioengineering Institute, China) following the manufacturer’s instructions.

### Statistical analysis

Data were normally distributed and analyzed using Student’s *t*-test or one-way analysis of variance as appropriate, with Tukey’s honestly significant difference *post hoc* test used to identify significant group differences. A significance threshold of p < 0.05 was applied. Results are presented as mean ± standard error of the mean. Graphs were generated using GraphPad Prism (v 8.0.0, GraphPad Software, San Diego, USA).

## RESULTS

### Growth and muscle development

**Figure 1:**
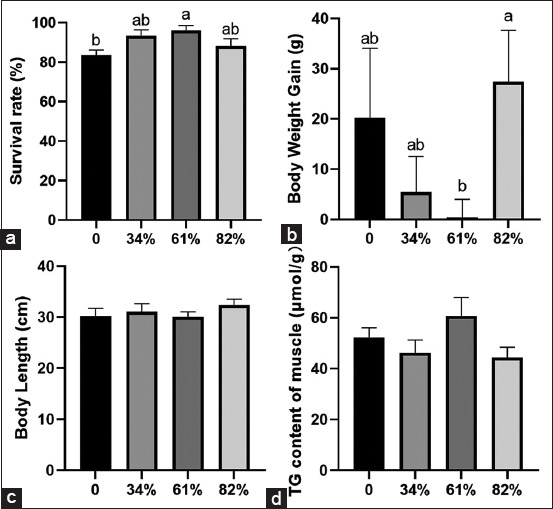
Replacing marine fish with BSFL fermented homogenate affects the growth performance of Asian swamp eels. (a) Survival rate, n = 9; (b) body weight gain, n = 9; (c) body length, n = 9; and (d) TG content of muscle, n = 6. Data presented are mean ± standard error of the mean and means with the same letter are not significantly different. Significant differences were accepted at p < 0.05. BSFL=Black soldier fly larvae.

**Figure 2:**
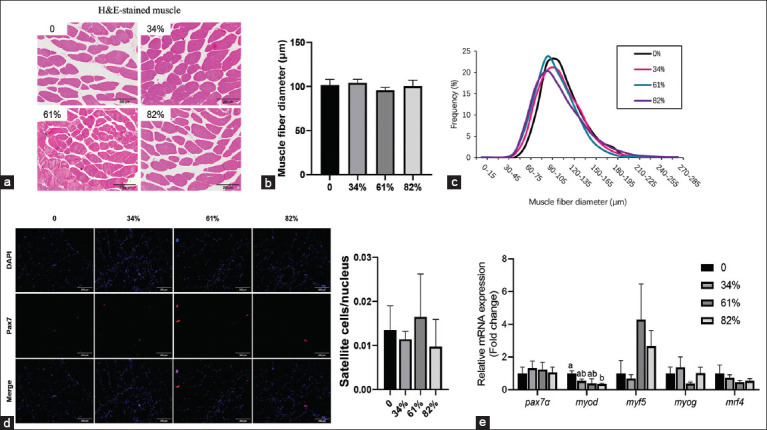
Replacing marine fish with BSFL fermented homogenate on the muscle development of Asian swamp eels. (a) Representative images of H&E-stained muscle (scale bar = 200 µm); (b) the average diameter of muscle fibers, n = 6; (c) distribution of the size of diameter of the muscle fibers, n = 6; (d) immunofluorescence-stained of satellite cells (Pax7+) in the muscle tissue, and the ratio of satellite cells to nucleus; and (e) the expression of myogenic genes in the muscle, n = 6. Data presented are mean ± standard error of the mean and means with the same letter are not significantly different. Significant differences were accepted at p < 0.05. BSFL=Black soldier fly larvae, H&E=Hematoxylin-eosin.

ASEs in the BSFL61 group exhibited a significantly higher survival rate compared to the control group (Figure 1a, p < 0.05). The inclusion of BSFL in the diet did not significantly affect body weight gain overall (Figure 1b); however, ASEs in the BSFL61 group gained significantly less weight than those in the BSFL82 group (p < 0.05). No significant differences were observed in body length (Figure 1c) or muscle TG content (Figure 1d) among the groups (p > 0.05). In addition, muscle fiber size did not differ significantly between groups (Figure 2a-c, p > 0.05), nor were there any notable differences in satellite cell counts (Figure 2d, p > 0.05). Further analysis of myogenic gene expression in muscle tissue revealed that ASEs in the BSFL82 group exhibited lower levels of *myod* expression compared to the control group (Figure 2e, p > 0.05).

### Hepatic health

**Figure 3:**
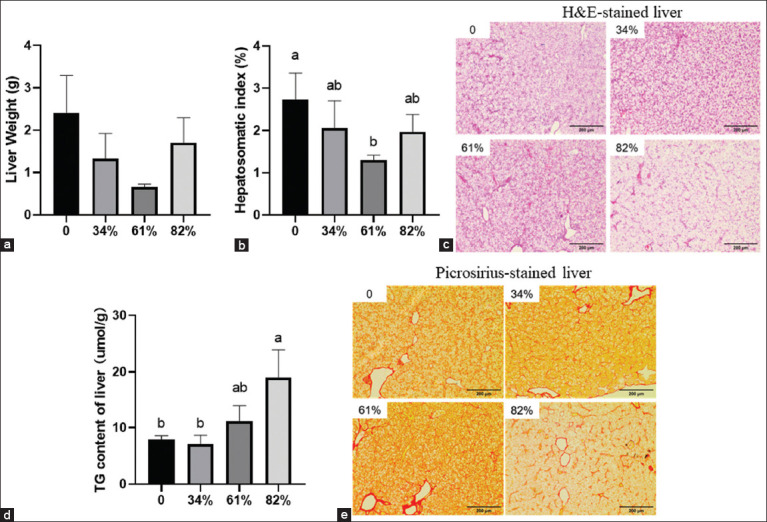
Replacing marine with BSFL fermented homogenate on the hepatic health of Asian swamp eels. (a) Liver weight, n = 9; (b) hepatosomatic index (%), n = 9; (c) representative images of H&E-stained liver (scale bar = 200 µm); (d) triglyceride content in the liver, n = 6; and (e) representative images of Picrosirius-stained liver (scale bar = 200 µm). Data presented are mean ± standard error of the mean and means with the same letter are not significantly different. Significant differences were accepted at p < 0.05. BSFL=Black soldier fly larvae, H&E=Hematoxylin-eosin.

ASEs in the BSFL61 group showed a trend toward lower liver weight (Figure 3a, p = 0.06) and had a significantly reduced hepatosomatic index compared to the control group (Figure 3b, p < 0.05). Notably, the BSFL82 group demonstrated a higher degree of fat accumulation in the liver relative to the control (Figure 3c and d, p < 0.05), whereas muscle TG content remained unaffected (Figure 1d, p > 0.05). This hepatic lipid accumulation is likely attributable to the higher crude fat (EE) content in the fermented BSFFH (Table 1), which resulted in increased EE content in the BSFL82 diet (Table 2). As the liver serves as the central organ for lipid metabolism [53], this led to increased hepatic fat storage without altering fat content in muscle tissue. Picrosirius red staining, which identifies collagen fibers indicative of tissue fibrosis [54], revealed that the BSFL82 group exhibited less hepatic fibrosis than the control group (Figure 3e).

### Intestinal development

**Figure 4:**
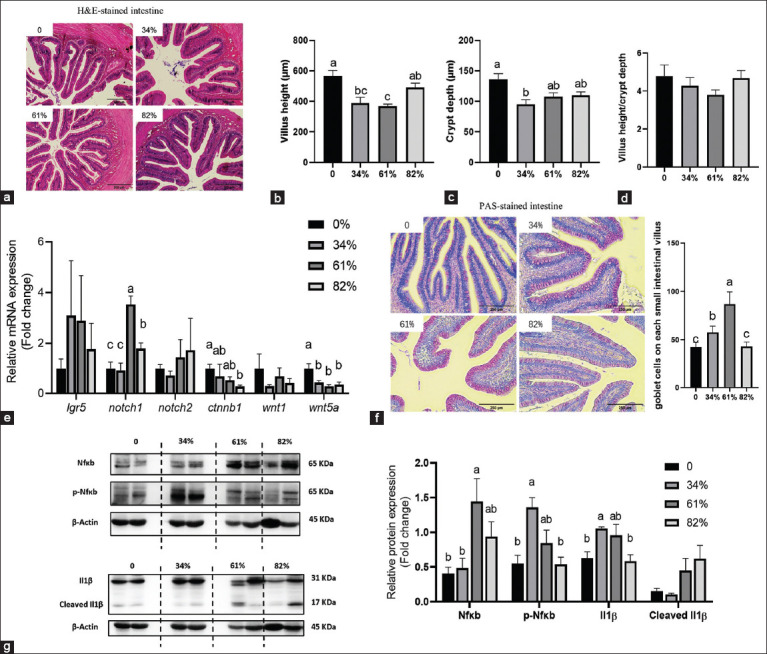
Replacing marine with BSF fermented homogenate on the intestine development of Asian swamp eels. (a) Representative images of H&E-stained intestine (scale bar = 200 µm); (b) Villus height of the intestine, n = 9; (c) crypt depth of the intestine, n = 9; (d) the ratio of Villus height/crypt depth, n = 9; (e) the expression of genes related to intestine epithelium development, n = 6; (f) representative images of PAS-stained intestine (scale bar = 100 µm); and (g) Western blot bands and quantification of NFκB, p-NFκB, IL-1β and cleaved IL-1β in the intestine, n = 4. Data presented are mean ± standard error of the mean and means with the same letter are not significantly different. Significant differences were accepted at *P* < 0.05. NFκB=Nuclear factor-kappa B, p-NFκB=Phospho nuclear factor-kappa B, IL-1β=Interleukin-1 beta, H&E=Hematoxylin-eosin, PAS=Periodic acid-Schiff.

Compared to the control group, the intestinal villus height was significantly reduced in both the BSFL34 and BSFL61 groups (Figure 4a and b), and crypt depth was significantly decreased in BSFL34 (Figure 4a and c) (P < 0.05). Although the villus height-to-crypt depth ratio in BSFL61 was also lower, the difference was not statistically significant (Figure 4d, p > 0.05). Moreover, BSFL inclusion led to upregulation of notch1 expression in both BSFL61 and BSFL82, while ctnnb1 was downregulated in BSFL82 and wnt5a was downregulated in all treatment groups (Figure 4e, p < 0.05). PAS staining further revealed increased goblet cells in the intestines of ASEs fed fermented BSFL (Figure 4f). In addition, the expression of inflammatory markers, including NfκB in BSFL61 and phosphorylated NfκB and IL-1β in BSFL34, was elevated (Figure 4, p < 0.05).

## DISCUSSION

As a protein-rich feed resource, BSFL serve as an effective substitute for fish meal [55–58]. However, careful consideration must be given to the potential nutritional implications associated with its high fat and chitin content. Numerous studies have reported no adverse effects on the growth of fish species such as Nile tilapia and Japanese seabass [59–61], and even enhancements in growth performance in species such as ASE and Siberian sturgeon have been documented with BSFL inclusion [30, 31]. In the present study, partial replacement of marine fish with fermented BSFL in ASE diets did not significantly affect body weight gain compared to the control group; however, ASEs in the BSFL61 group showed lower weight gain than those in the BSFL82 group. Recent findings suggest that BSFL contains approximately 9% chitin [62], a well-recognized antinutritional factor. Dietary chitin levels exceeding 1% have been shown to negatively impact fish growth and nutrient utilization [63, 64]. In this context, replacing 34% or 61% of marine fish with fermented BSFL resulted in a reduction in intestinal villus height, and the increased number of goblet cells in BSFL61 suggests enhanced mucin secretion [65]. These findings imply that fermented BSFL may have impaired nutrient digestion and absorption in ASE, although growth performance was not markedly compromised. The survival rate of ASEs remained within the normal range (80%–90%) throughout the 90-day trial [30, 50, 52, 66].

Goblet cells play key roles in mucin secretion, formation of a protective mucus barrier [67], and production of antimicrobial proteins, chemokines, and cytokines that modulate immune responses [68]. The observed increase in goblet cell numbers in the BSFL61 group may represent a protective adaptation. In addition, elevated NfκB levels in BSFL61 and increased IL-1β expression in BSFL34 indicate activated intestinal immune responses, which may have contributed to enhanced resistance against enteric pathogens and, in turn, improved survival. BSFL is known to contain AMPs [40–42] and MCFAs [38], which possess immunoregulatory and antimicrobial properties. Previous studies have shown that BSFL supplementation improves intestinal health and microbial balance in aquatic species such as rice field eel [30, 39], Atlantic salmon [14], and golden pompano [69]. However, the heightened immune activity observed in BSFL34 and BSFL61 may have increased the energy expenditure of ASEs. This energy diversion, potentially coupled with a reduction in absorptive and digestive epithelial cells due to an expanded goblet cell population, may explain the modest reduction in growth performance. Notably, the observed upregulation of *notch1* and downregulation of *wnt5a* may reflect a compensatory mechanism to stimulate absorptive cell differentiation in response to their relative deficiency [70, 71].

Full-fat BSFL has a fat content ranging from 294 g/kg to 515.3 g/kg on a dry matter basis, which is substantially higher than that of fishmeal [8]. BSFL fat predominantly comprises saturated fatty acids (up to 76%), with lower levels of monounsaturated and polyunsaturated fatty acids [72]. While MCFAs in BSFL contribute to antimicrobial defense, the broader metabolic implications of excessive BSFL fat should not be overlooked. In this study, ASEs fed fermented BSFL exhibited elevated hepatic TG levels, likely due to the higher dietary crude fat content in BSFL-substituted feeds. Excessive hepatic lipid accumulation can lead to metabolic disorders [73]. The BSFL61 group showed increased hepatic fibrosis, whereas the BSFL82 group exhibited lower fibrosis levels despite having the highest liver fat content. This suggests that fat accumulation may precede fibrosis development [74]; however, further research is needed to determine whether fermented BSFL provides hepatoprotective compounds.

In conclusion, the inclusion of fermented BSFL at a substitution level of 61% improved ASE health by enhancing intestinal immunity and survival during the 90-day feeding period. Nevertheless, due to the short duration of the study, the long-term health implications of BSFL inclusion remain uncertain. Since higher inclusion levels (e.g., BSFL82) elevated hepatic lipid content, extended feeding may adversely affect liver health. In this study, BSFL did not significantly impact overall ASE productivity. The lower weight gain observed in the BSFL61 group relative to BSFL82 may be attributed to increased energy expenditure due to heightened gut immune activity. In practical aquaculture settings, it may be possible to sustain both weight gain and immune function in ASE by slightly reducing the level of BSFL inclusion. In addition, since BSFL are typically reared on food waste [1] and animal manure [2–5], and fermented BSFL can be stored and transported without cold-chain requirements, their large-scale adoption in aquaculture could reduce environmental impact as well as feed transportation and storage costs.

## CONCLUSION

This study demonstrates that fermented BSFL can serve as a sustainable and effective partial replacement for marine fish in the diet of ASE (*M. albus*). The 61% substitution level notably improved survival rates, enhanced intestinal immune responses – evidenced by increased goblet cell numbers and upregulation of immune-related markers – and did not compromise overall growth performance. Furthermore, fermented BSFL inclusion resulted in favorable modulation of hepatic and intestinal gene expression and maintained stable muscle development across treatment groups.

A key strength of this study lies in its comprehensive assessment of physiological, histological, and molecular responses to BSFL inclusion, providing multi-layered evidence for the nutritional and immunological benefits of this alternative feed source. The fermentation process, by preserving bioactive compounds and improving digestibility, adds further value to BSFL as a viable aquafeed ingredient, especially in regions where cold-chain logistics are limiting.

However, several limitations should be acknowledged. Although short-term feeding (90 days) yielded promising results, the long-term effects of elevated hepatic lipid accumulation – particularly at higher BSFL inclusion levels (82%) – remain unclear. The study also did not evaluate reproductive performance, feed conversion efficiency, or sensory and nutritional quality of ASE flesh, which are critical for commercial adoption.

Future studies should explore the effects of prolonged BSFL feeding on liver function, metabolic health, and overall productivity. Investigations into optimizing the fermentation process to reduce fat content while preserving bioactivity could further enhance its nutritional profile. Moreover, expanding research to include economic analysis and environmental impact assessments will support broader application and policy development for sustainable aquaculture practices.

In summary, fermented BSFL represents a promising protein source for aquafeeds, offering nutritional, environmental, and logistical advantages. Its strategic inclusion in ASE diets could contribute to more resilient and sustainable aquaculture systems, which provided that inclusion levels are carefully optimized to balance immune enhancement with metabolic health.

## AUTHORS’ CONTRIBUTIONS

YX: Designed the study, animal husbandry, fermentation experiments, sample processing, tissue sectioning, H&E staining, immunohistology staining, PAS staining, picrosirius red staining, RNA extraction, qRT-PCR, protein extraction, western blotting, TG analysis, statistical analysis of data, and manuscript writing. SG: Designed the study, animal husbandry, fermentation experiments, sample processing, tissue sectioning, H&E staining, and statistical analysis of data. YL, GT, and LZ: Fermentation experiments. ZT and XZ: Animal husbandry. KX, WN, XL, and JX: Collection of samples. BW: Experimental design, process supervision, and manuscript revision. All authors have read and approved the final manuscript.
